# Down-regulation of lncRNA UCA1 enhances radiosensitivity in prostate cancer by suppressing EIF4G1 expression via sponging miR-331-3p

**DOI:** 10.1186/s12935-020-01538-8

**Published:** 2020-09-11

**Authors:** Minhua Hu, Jincheng Yang

**Affiliations:** 1 Department of Nursing College, Xi’an Medical University, Xi’an, 710021 Shaanxi Province China; 2 Department of Urology Surgery, The First People’s Hospital of Yinchuan, No. 4, Liqun West Street, Xingqing District, Yinchuan, 750004 Ningxia China

**Keywords:** UCA1, Prostate cancer, miR-331-3p, EIF4G1, Radioresistance

## Abstract

**Background:**

We aimed to explore the role of long noncoding RNA urothelial carcinoma-associated 1 (lncRNA UCA1) and its underlying mechanism in the radioresistance of prostate cancer (PCa).

**Methods:**

QRT-PCR was conducted to measure the expression of UCA1, microRNA-331-3p (miR-331-3p) and eukaryotic translation initiation factor 4 gamma 1 (EIF4G1) in PCa tissues and cells. The relative protein level was determined by western blot assay. Cell proliferation and apoptosis were detected by MTT, colony formation assay, and flow cytometry, respectively. The target interaction between miR-331-3p and UCA1 or EIF4G1 was predicted through bioinformatics analysis, and verified by dual-luciferase reporter gene assay system.

**Results:**

The high levels of UCA1 and EIF4G1 as well as the low level of miR-331-3p were observed in PCa tissues and cell lines. UCA1 and EIF4G1 expression were significantly upregulated by Gy radiation treatement. UCA1 or EIF4G1 knockdown repressed cell growth and enhanced cell apoptosis in 22RV1 and DU145 cells under radiation. Moreover, overexpression of EIF4G1 abolished UCA1 knockdown-induced effect on 6 Gy irradiated PCa cells. UCA1 sponged miR-331-3p to regulate EIF4G1 expression.

**Conclusions:**

LncRNA UCA1 deletion suppressed the radioresistance to PCa by suppressing EIF4G1 expression via miR-331-3p. UCA1 acted as a potential regulator of radioresistance of PCa, providing a promising therapeutic target for PCa.

## Highlights

1. UCA1 and EIF4G1 levels were increased in PCa tissues and cells.
2. Downregulation of UCA1 enhanced radiosensitivity in PCa.
3. MiR-331-3p was decreased in PCa tissues and cells.
4. MiR-331-3p was a target for UCA1 and it could regulate the expression of EIF4G1.
5. UCA1 downregulation facilitated radiosensitivity via miR-331-3p/EIF4G1 axis in vitro.

## Background

Prostate cancer (PCa) is one of the most common malignant tumors worldwide [1]. Radiotherapy is a choice for the regionally unresectable advanced PCa patients [2]. However, there are still some PCa patients presented uncontrollable or recurrent due to the radioresistance. Thus, it is urgent to explore the novel therapeutic strategy to enhance tumor radiosensitivity for the treatment of PCa patients.

Long non-coding RNAs (LncRNAs), as crucial modulators, participate in the initiation and development of many diseases, including cancers [3–5]. Urothelial cancer associated-1 (UCA1) was identified as an oncogene in various cancers [6–8]. For example, overexpression of UCA1 promoted the progression of breast cancer [9]. Similarly, abundance of UCA1 accelerated cell metastasis via Hippo pathway in pancreatic cancer [10]. Enhanced expression of UCA1 contributed to the radioresistance to PCa, and high expression of UCA1 was observed in the irradiation resistance DU145 cells [11]. Thus, we aimed to investigate the role of UCA1 and its underlying mechanism in radioresistant PCa cells.

MicroRNAs (miRNAs) are small non-coding RNAs with 19–25 endogenous nucleotides in length [12–14]. Generally, miRNAs acted as tumor promoters or suppressors to regulate cancer cell cycle, survival, differentiation, metastasis, epithelial-mesenchymal transition (EMT), autophagy and apoptosis by interacting with the messenger RNAs (mRNAs) [15–17]. Dysregulation of miR-331-3p was observed in various cancers [18–20]. StarBase v3.0 predicted that miR-331-3p was a potential target of UCA1. More importantly, miR-331-3p repressed cell proliferation and migration in PCa cells [21–24]. Moreover, miRNAs were reported to regulate the radiosensitivity in PCa, such as miR-18a and miR-205 [25, 26]. However, the regulatory role of miR-331-3p in the radiosensitivity of PCa is still unclear.

Eukaryotic translation initiation factor 4 gamma 1 (EIF4G1) was located on chromosome 3q27.1. The interaction between EIF4G and EIF4E promotes EIF4F complexes formation, which played a vital pattern in tumorigenesis [27, 28]. Moreover, EIF4G1 was found to be overexpressed in several solid tumors [29–36], such as hypopharyngeal cancer, cervical carcinoma, breast cancer, multiple myeloma, lung cancer, ovarian cancer, and PCa. However, the role of EIF4G1 in radiotherapy resistance has not been studied in PCa. Therefore, it was of significance to reveal the role of EIF4G1 in the radiosensitivity of PCa.

In this study, we investigated the role of UCA1 in radiotherapy resistance of PCa progression in vitro. Moreover, the regulatory mechanism of UCA1/miR-331-3p/EIF4G1 axis in the radioresistant PCa cells was explored.

## Materials and methods

### Patient samples

Tumor tissues (n = 40) and the paired adjacent normal tissues (n = 40) were collected from PCa patients by surgery in Xi’an medical university. All patients signed the written informed consents. Our experimental protocols were approved by Ethics Committee of Xi’an medical university.

### Cell culture and transfection

Human prostatic epithelial cells (RWPE1) and two PCa cell lines (22RV1 and DU145) were purchased from Biomedical Science cell bank (Shanghai, China). Dulbecco’s Modified Eagle Medium (DMEM, Gibco, Carlsbad, CA, USA) was used to culture cells. EIF4G1 overexpression vector was obtained by cloning the sequence of EIF4G1 into pcDNA3.1 (Invitrogen, Carlsbad, CA, USA), termed as pcDNA-EIF4G1. One day before transfection, PCa cells were seeded into a 12-well plate. 0.2 µg of EIF4G1 was transfected in PCa cells (5 × 10^5^ cells/well) with 0.5 µL of Lipofectamine 2000 reagent (Invitrogen). Moreover, those oligonucleotides included that small interfering RNA (siRNA) targeting UCA1 (si-UCA1#1, si-UCA1#2, and si-UCA1#3), siRNA targeting EIF4G1 (si-EIF4G1#1, si-EIF4G1#2, si-EIF4G1#3), siRNA negative control (si-NC) were synthesized by Genepharma (Shanghai, China), and miR-331-3p mimics (miR-331-3p), miR-331-3p inhibitor (anti-miR-331-3p) and the corresponding miRNA negative controls (miR-NC and anti-miR-331-3p) were purchased from RIBOBIO (Guangzhou, China). Then, cells were transfected with 0.5 µg of the aforementioned oligonucleotides using 0.6 µL of Lipofectamine 2000 (Invitrogen). The sequences of siRNAs were as follows:

si-UCA1#1: GUGAAGACAAUCAACUCAAUU, si-UCA1#2: CCAGCCAUACAGGACCAGAUU, si-UCA1#3: GAGCCGAUCAGACAAACAAUU, si-EIF4G1#1: CCTAGTGCCTCTGAGAATT, si-EIF4G1#2: CCACTCATCTTATAGCTTT, si-EIF4G1#3: GCAGTTATAGGTGGGACAT.

### Quantitative real-time polymerase chain reaction (qRT-PCR)

The tissues and cells were lysed using TRIzol reagent (Invitrogen, Carlsbad, CA, USA) to isolate total RNA. The cDNA for UCA1, miR-331-3p and EIF4G1 was synthesized by All-in-One™ Kit (FulenGen, Guangzhou, China). QRT-PCR was conducted by SYBR green (Applied Biosystems, Foster City, CA, USA). Glyceraldehyde-3-phosphate dehydrogenase (GAPDH) and U6 snRNA were served as internal references. The primers for UCA1, miR-331-3p, EIF4G1, GAPDH and U6 were listed as blow: UCA1, 5′-CCACACCCAAAACAAAAAATCT-3′ (sense) and 5′-TCCCAAGCCCTCTAACAACAATGAC-3′ (antisense); miR-331-3p, 5′-CAACAAAATCACTAGTCTTCCA-3′ (sense) and 5′-TGGAAGACTAGTGATTTTGTTG-3′(antisense); EIF4G1, 5′-CATTGGCTGCCTTGGGACTA-3′ (sense) and 5′-ATGCAAGGTTCCAAGGGTCC-3′ (antisense); GAPDH, 5′-AGGTCGGTGTGAACGGATTTG-3′ (sense) and 5′-GGGGTCGTTGATGGCAACA-3′ (antisense); U6, 5′-ACCCTGAGAAATACCCTCACAT-3′ (sense) and 5′-GACGACTGAGCCCCTGATG-3′ (antisense). The relative expression was determined by the 2^−ΔΔCt^ method.

### Western blot

Total protein was collected from tissues and cells using RIPA buffer (Solarbio). 20 µg proteins was separated by SDS-PAGE, and then transferred onto polyvinylidene fluoride (PVDF) membranes. Then, membranes were incubated with the primary antibodies at 4℃ overnight. Following 2-h incubation with goat anti-rabbit IgG H&L (HRP) antibody (1:1000; ab205718, Abcam, Cambridge, UK), the chemiluminescence was detected using an ECL detection kit (Beyotime, Shanghai, China). The primary antibodies used in this study included anti-EIF4G1 (ab2609, 1:1000, Abcam, Cambridge, MA, USA), anti-CyclinD1 (ab134175, 1:1000, Abcam), anti-B-cell lymphoma-2 (Bcl-2, ab196495, 1:1000, Abcam), anti-Bax (Bcl-2-associated X protein, ab53154, 1:1000, Abcam), and anti-GAPDH (ab9485, 1:2500, Abcam).

### Cell viability assays

22RV1 and DU145 cells were seeded in a 96-well plate for 24 h and then exposed to 6 Gy radiation. At 0, 24, 48, and 72 h after radiotherapy, cell viability was determined by the MTT Kit (Beyotime, Shanghai, China). 10 µL MTT (Beyotime, Shanghai, China) was added into each well and incubated for 4 h. Then, cells were dissolved in dimethyl sulfoxide (DMSO; Sigma, St. Louis, MO, USA) for 2 h. Finally, the optical density (OD) value at 490 nm was determined by a spectrophotometer.

### Colony formation assay

Transfected 22RV1 and DU145 cells were irradiated with different Gy radiation dose ranges (0, 2, 4, 6 and 8 Gy). Then, the colonies were fixed with methanol, stained with 1% crystal violet solution (Sigma, St. Louis, MO, USA). Finally, the number of colonies (more than 50 cells) was counted under a microscope.

### Flow cytometry

Transfected 22RV1 and DU145 cells were plated on 24-well plates for 48 h. Subsequently, the cells were collected and stained with 5 µL fluorescein isothiocyanate tagged Annexin V (Annexin V-FITC)/propidium iodide (PI) (Invitrogen). Finally, the apoptotic rate was detected and analyzed by a flow cytometer.

### Dual-luciferase reporter assay

Wild type UCA1 (WT-UCA1) (containing the complementary binding sites) and mutant type UCA1 (MUT-UCA1) luciferase vectors were constructed. Meanwhile, wild type EIF4G1 (3′-UTR-WT EIF4G1) and mutant type EIF4G1 (3′-UTR-MUT EIF4G1) luciferase vectors were constructed. Then, Those vectors were co-transfected with miR-331-3p or miR-NC into 22RV1 and DU145 cells. Finally, the luciferase activity was determined using Dual-Lucy Assay Kit (Promega, Madison, WI, USA).

### Statistical analysis

Data from at least three repeated experiments were exhibited as mean ± standard deviation (SD). The difference analyses were analyzed using one-way analysis of variance (ANOVA) or Student’s *t*-test. A value *P* < 0.05 was regarded as statistically significant.

## Results

### UCA1 was positively related to EIF4G1 expression in PCa tissues

**Fig. 1:**
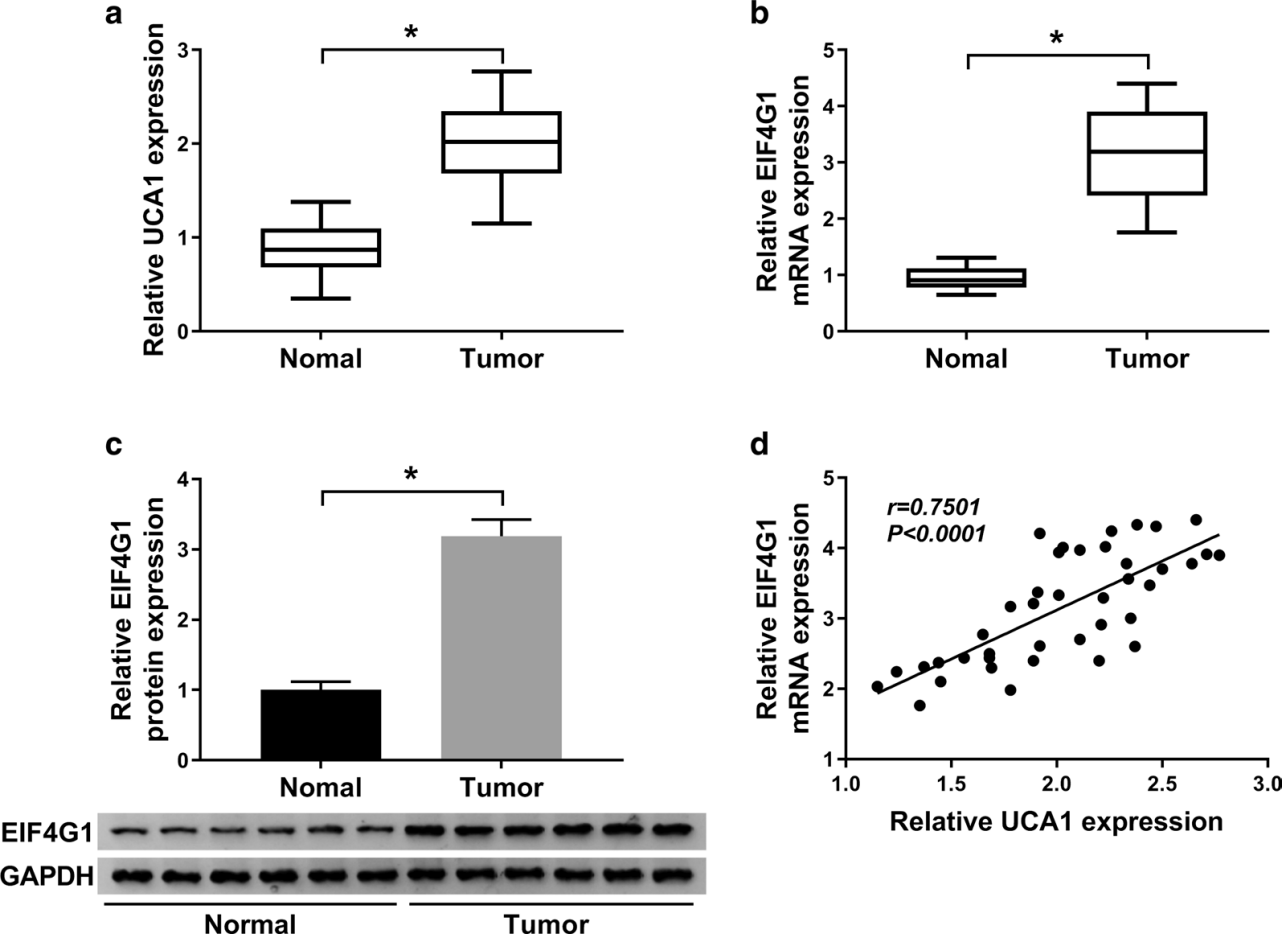
UCA1 was positively related to EIF4G1 expression in PCa tissues. **a**, **b** The expression of UCA1 (**a**) and EIF4G1 (**b**) was measured by qRT-PCR analysis in PCa tissues (n = 40) or adjacent non-cancer tissues (n = 40). **c** The protein level of EIF4G1 was detected by western blot in PCa tissues (n = 40) or adjacent non-cancer tissues (n = 40). **d** Correlation between UCA1 and EIF4G1 expression in PCa tissues was analyzed. ^*^P < 0.05

As shown in Fig. 1a–c, the expression level of UCA1 (Fig. 1a) was markedly enhanced in PCa tissues. EIF4G1 mRNA (Fig. 1b) and protein (Fig. 1c) expression were upregulated in PCa tissues. Moreover, we observed a positive relationship between UCA1 level and EIF4G1 expression in PCa tissues (Fig. 1d). These results demonstrated that UCA1 and EIF4G1 might be involved in the pathogenesis of PCa.

### Radiation treatment increased UCA1 expression and EIF4G1 protein level in PCa cells

**Fig. 2:**
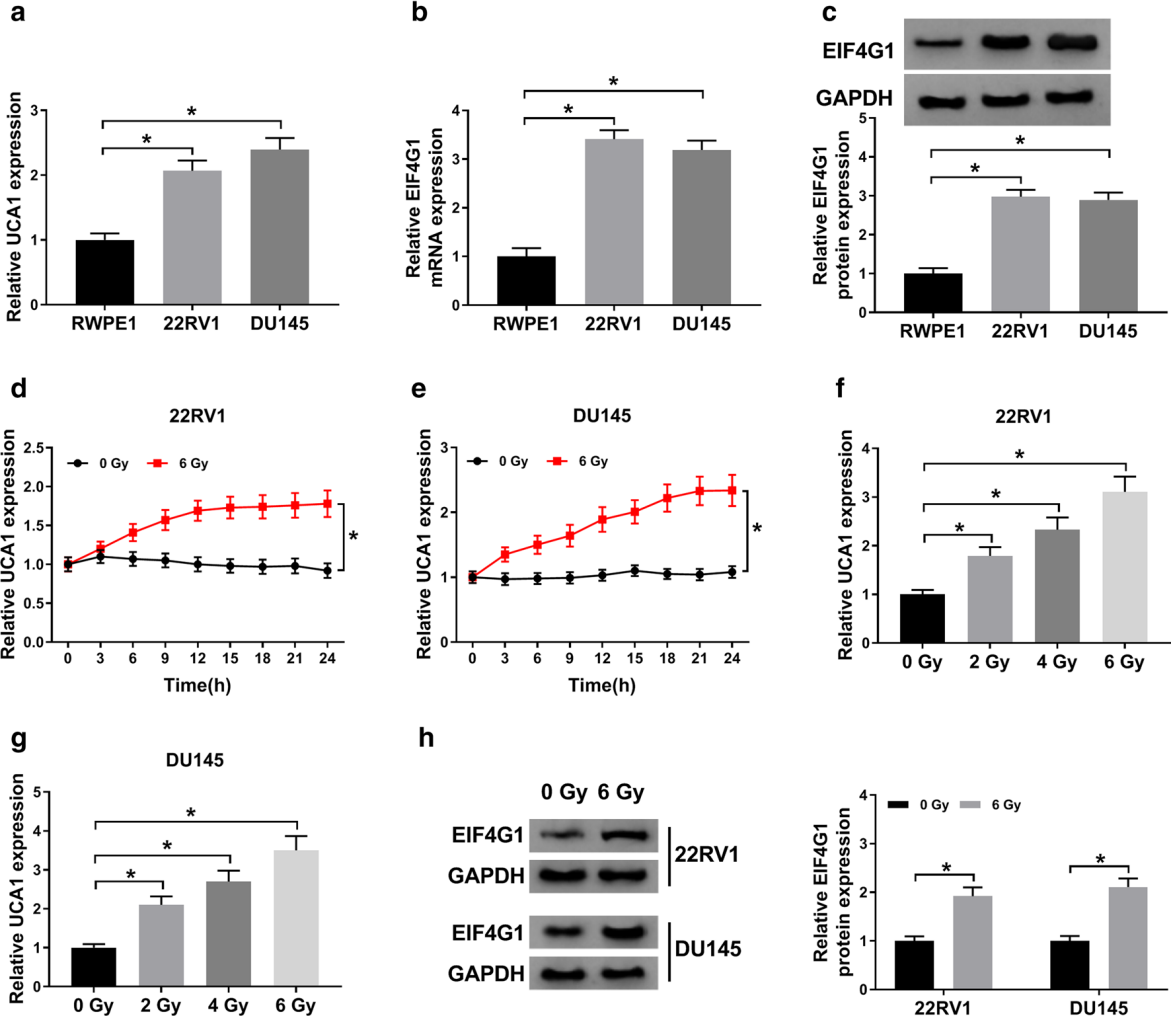
Radiation treatment increased UCA1 expression and EIF4G1 protein level in PCa cells. **a** QRT-PCR analysis was conducted to detect UCA1 expression in PCa cell lines (22RV1 and DU145 cells) and human normal prostatic epithelial cells RWPE1. **b**, **c** The mRNA (**b**) or protein (**c**) level of EIF4G1 was determined by qRT-PCR or western blot in 22RV1 and DU145 cells, respectively. **d**, **e** UCA1 expression was detected in 22RV1 (**d**) and DU145 (**e**) cells every 3 h after 0 or 6 Gy radiation treatment. **f**, **g** UCA1 expression was measured in 22RV1 (**f**) and DU145 (**g**) cells after 0, 2, 4, 6 Gy radiation treatment for 24 h. **h** EIF4G1 protein level was upregulated after 22RV1 and DU145 cells treated with 6 Gy radiation for 24 h. ^*^P < 0.05

UCA1 was significantly elevated in PCa cells (22RV1 and DU145) compared with RWPE1 cells (Fig. 2a). Similarly, EIF4G1 mRNA (Fig. 2b) and protein (Fig. 2c) levels were dramatically upregulated in 22RV1 and DU145 cells. Then, 22RV1 and DU145 cells were treated with 6 Gy radiation to determine the effect of radiation treatment on UCA1 and EIF4G1 expression. The data showed that radiation treatment markedly enhanced UCA1 expression in (Fig. 2d–g). Moreover, EIF4G1 protein level was promoted by radiation exposure in both 22RV1 and DU145 cells (Fig. 2h). Overall, these data indicated that radiation treatment promoted UCA1 and EIF4G1 expression in PCa cells.

### UCA1 knockdown enhanced the radiosensitivity of PCa cells

**Fig. 3:**
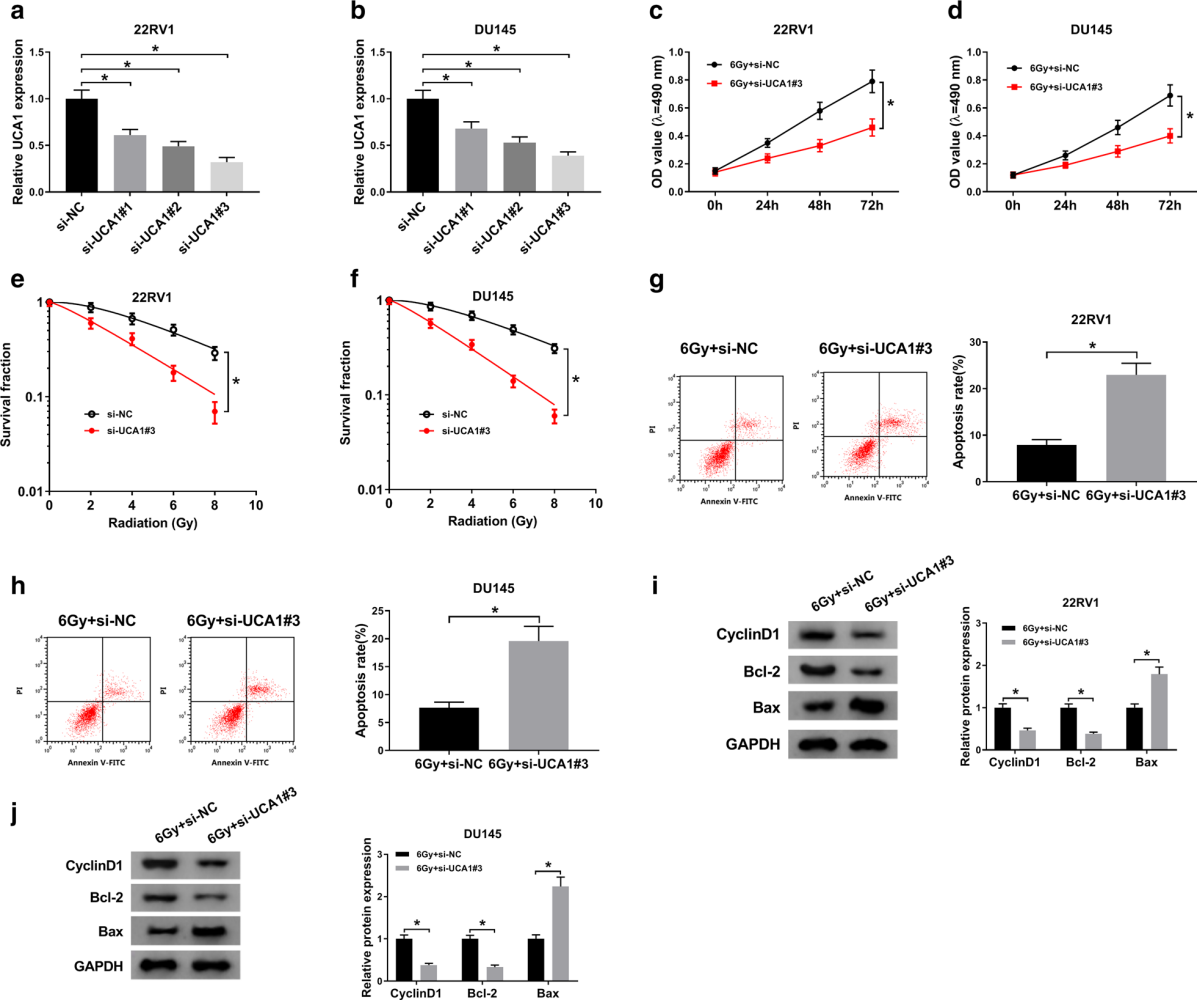
UCA1 knockdown enhanced the radiosensitivity of PCa cells. **a**, **b** The relative UCA1 expression was detected by qRT-PCR in 22RV1 (**a**) and DU145 (**b**) cells transfected with si-UCA1#1, si-UCA1#2, si-UCA1#3, or si-RNA. **c–j** 2RV1 and DU145 cells were transfected with si-NC or si-UCA1#3. **c**, **d** Cell viability was detected in transfected 22RV1 (**c**) and DU145 (**d**) cells at 0, 24, 48, and 72 h after Gy irradiation. **e**, **f** Transfected 22RV1 (**e**) and DU145 (**f**) cells were subjected to 0, 2, 4, 6 and 8 Gy irradiation. After 2 weeks, the colony survival fractions were measured. **g**, **h** Cell apoptosis was determined in transfected 22RV1 and DU145 cells at 24 h after 6 Gy radiotherapy. **i**, **j** The protein expression of Cyclin D1, Bcl-2, and Bax was examined in transfected 22RV1 (**i**) and DU145 (**j**) cells after 6 Gy irradiation. ^*^P < 0.05

The colony survival assay and anti-apoptosis activities of cancer cells were considered to be closely related to radioresistance [37–41]. To explore whether UCA1 could affect the radiosensitivity of PCa cells, loss-of function assay was carried out. Among the designed three siRNAs (si-UCA1#1, siUCA1#2 and si-UCA1#3), si-UCA1#3 showed the highest knockdown efficiency in 22RV1 (Fig. 3a) and DU145 (Fig. 3b) cells, thus si-UCA1#3 was chose for the subsequent experiments. MTT assay indicated that UCA1 deletion markedly suppressed cell growth of 6 Gy irradiated 22RV1 and DU145 cells (Fig. 3c, d). Moreover, UCA1 downregulation significantly repressed the survival fractions in 6 Gy irradiated 22RV1 and DU145 cells (Fig. 3e, f). Flow cytometry analysis revealed that UCA1 silencing significantly enhanced the apoptosis rate of 6 Gy irradiated 22RV1 and DU145 cells (Fig. 3g, f). Western blot result showed that decrease of UCA1 significantly inhibited the expression of CyclinD1 and Bcl-2, but promoted Bax expression in 6 Gy irradiated 22RV1 and DU145 cells (Fig. 3i, j). Collectively, UCA1 knockdown enhanced the radiosensitivity of 22RV1 and DU145 cells.

### EIF4G1 knockdown facilitated the radiosensitivity in PCa cells

**Fig. 4:**
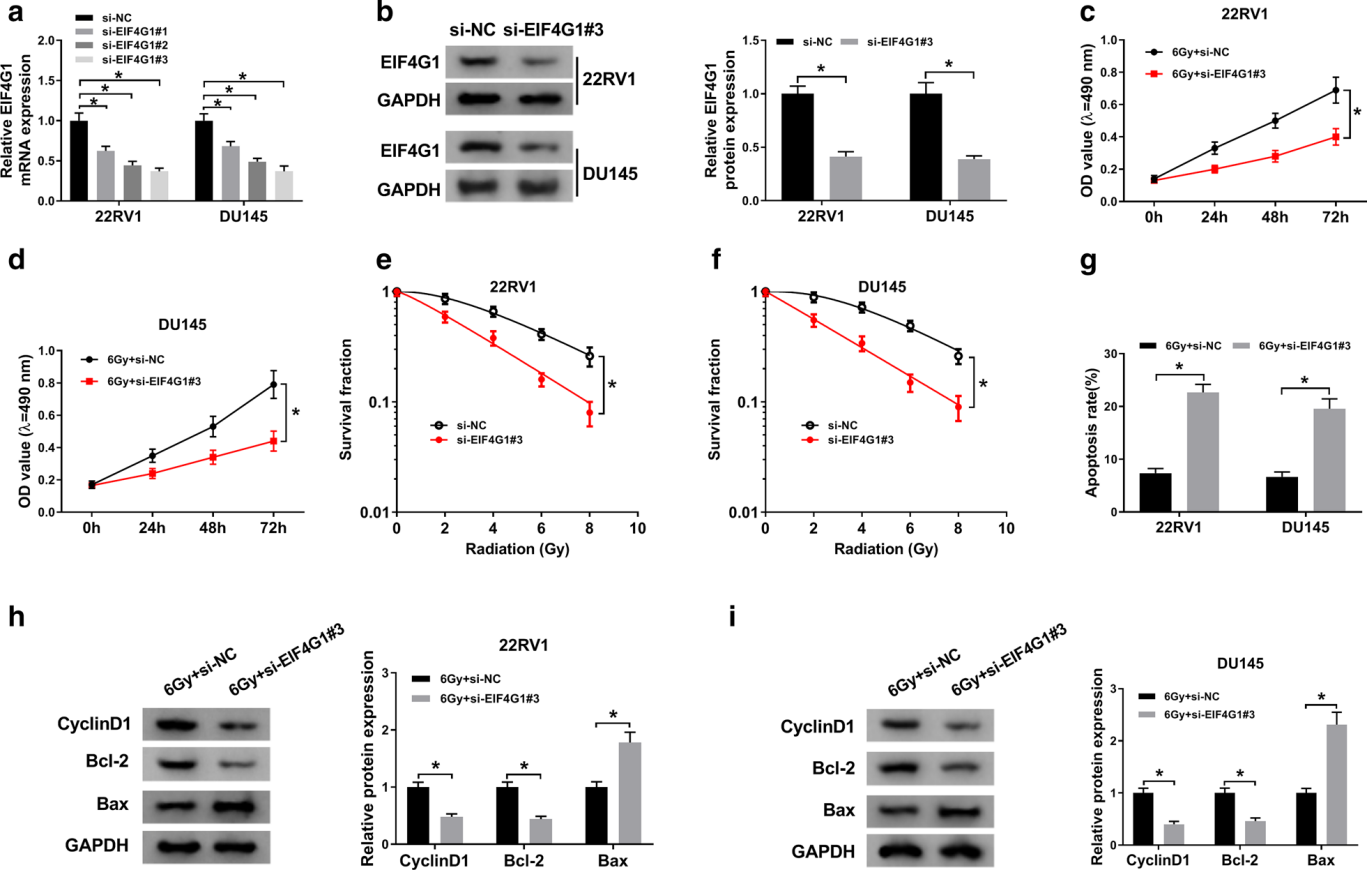
EIF4G1 knockdown facilitated the radiosensitivity in PCa cells. **a**, **b** The relative mRNA and protein expression of EIF4G1 were determined in 22RV1 and DU145 cells transfected with si-EIF4G1#1, si-EIF4G1#2, si-EIF4G1#3 or si-NC by qRT-PCR and western blot, respectively. **c**–**i** Cells were transfected with si-NC or si-EIF4G1#3. **c**, **d** MTT assay was used to examine cell viability of transfected 22RV1 (**c**) and DU145 (**d**) cells after 6 Gy irradiation. **e**, **f** The colony survival fractions were measured in transfected 22RV1 (**e**) and DU145 (**f**) cells subjected to irradiation. **g** Cell apoptosis was determined in transfected 22RV1 and DU145 cells after 6 Gy radiotherapy. **h**, **i** The protein expression of Cyclin D1, Bcl-2, and Bax was examined in transfected 22RV1 (**h**) and DU145 (**i**) cells after 6 Gy irradiation. ^*^P < 0.05

To explore whether EIF4G1 could affect the radiosensitivity in PCa cells, EIF4G1 was knocked down by transfecting si-EIF4G1 into PCa cells. Among the designed three siRNAs (si-EIF4G1#1, siEIF4G1#2 and si-EIF4G1#3), si-EIF4G1#3 was confirmed to possess the highest knockdown efficiency (Fig. 4a) and si-EIF4G1#3 was chose for further experiments (Fig. 4b). Our data demonstrated that EIF4G1 knockdown restrained cell viability of 22RV1 and DU145 cells under irradiation treatment compared with control group (Fig. 4c, d). Moreover, EIF4G1 silencing significantly repressed the survival fractions in 22RV1 and DU145 cells under irradiation treatment (Fig. 4e, f). Furthermore, EIF4G1 silencing enhanced the number of apoptotic cells in 22RV1 and DU145 cells under irradiation exposure (Fig. 4g). Western blot results showed that decrease of EIF4G1 weakened the expression of CyclinD1 and Bcl-2, but increased Bax expression in 22RV1 and DU145 cells under irradiation treatment (Fig. 4h, i). Overall, these data demonstrated that EIF4G1 silencing contributed to the radiosensitivity of PCa cells.

### Upregulation of EIF4G1 weakened the effect of UCA1 knockdown on the radiosensitivity of PCa cells

**Fig. 5:**
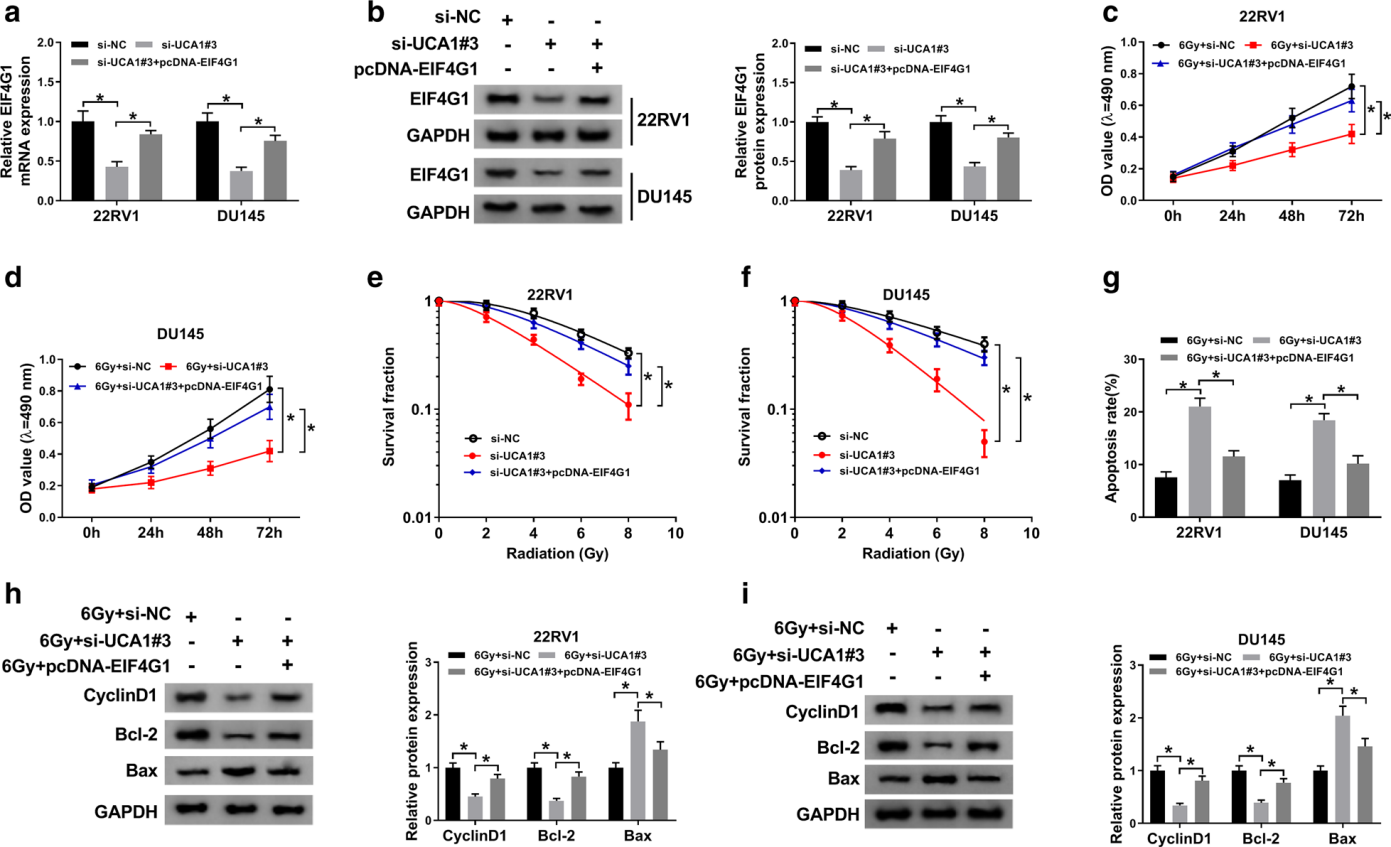
Upregulation of EIF4G1 weakened the effect of UCA1 knockdown on the radiosensitivity of PCa cells. 22RV1 and DU145 cells were transfected with si-UCA1 or co-transfected with si-UCA1 and pcDNA-EIF4G1. **a**, **b** The mRNA (**a**) and protein (**b**) expression of EIF4G1 were measured in PCa cells. **c**, **d** Cell proliferation was detected using MTT assay at the indicated times after 6 Gy irradiation treatment. **e**, **f** The clonogenic survival curve was established at day 14 after transfected cells received indicated Gy irradiation. **g** Cell apoptosis was measured by flow cytometry after radiotherapy. **h**, **i** Protein expression of Cyclin D1, Bcl-2, and Bax was detected in 22RV1 (**h**) and DU145 (**i**) cells exposure to radiotherapy. ^*^P < 0.05

As shown in Fig. 5a, b, UCA1 knockdown suppressed the mRNA and protein expression of EIF4G1, which was blocked by EIF4G1 overexpression. Moreover, the inhibition effects of UCA1 deletion on cell proliferation (Fig. 5c–f) and the promotion effect on cell apoptosis (Fig. 5g) were partially reversed by EIF4G1 upregulation in Gy irradiated 22RV1 and DU145 cells. Furthermore, pcDNA-EIF4G1 also inverted UCA1 deletion-caused decrease of CyclinD1 and Bcl-2 expression as well as increase of Bax expression in 22RV1 and DU145 cells under irradiation treatment (Fig. 5h, i). Taken together, knockdown of UCA1 enhanced the radiosensitivity of PCa cells by downregulating EIF4G1 expression.

### UCA1 regulated the expression of EIF4G1 via sponging miR-331-3p

**Fig. 6:**
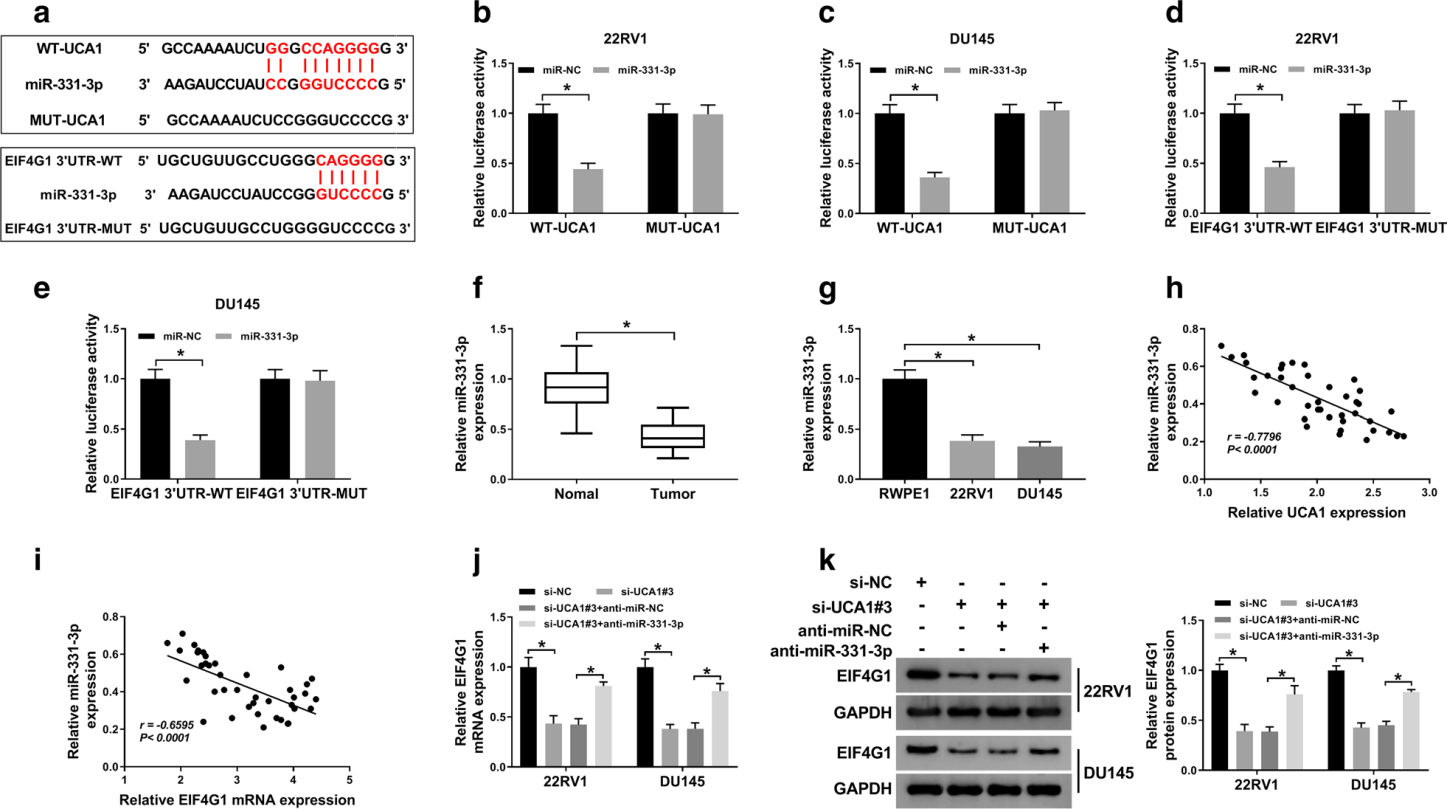
UCA1 regulated the expression of EIF4G1 via sponging miR-331-3p.** a** Bioinformatics (StarBase v3.0 software online) predicted the complementary binding sites between miR-331-3p and UCA1 or EIF4G1. **b**, **c** The direct combination between UCA1 and miR-331-3p in 22RV1 (**b**) and DU145 (**c**) was verified by dual-luciferase reporter assay. **d**, **e** The directive binding sequences between 3′UTR EIF4G1 and miR-331-3p in 22RV1 (**d**) and DU145 (**e**) were verified by dual-luciferase reporter assay. **f**–**g** QRT-PCR was used to evaluate the level of miR-331-3p in PCa tissues (**f**) and cells (**g**). **h**, **i** The correlation analysis between miR-331-3p and UCA1 or EIF4G1 was presented. **j**, **k** QRT-PCR or western blot assay was carried out to detect the mRNA (**j**) or protein (**k**) expression of EIF4G1 in PCa cells, respectively. ^*^P < 0.05

Previous researches reported that UCA1 could sponge several miRNAs to regulate RNA molecules, such as miR-145-5p [42], miR-124 [43], miR-200c [44], miR-185-5p [45], and miR-135a [46]. Thus, we hypothesized that UCA1 might regulate EIF4G1 expression by suppressing miRNA function. To confirm our hypothesis, starBase v3.0 online software (http://starbase.sysu.edu.cn/) was used to predict the potential targets of UCA1. StarBase v3.0 showed that miR-331-3p might be a potential target of UCA1, and EIF4G1 harbored the binding sites of miR-331-3p (Fig. 6a). As described in Fig. 6b, c, the luciferase activity was suppressed by miR-331-3p mimic treatment in wild-type UCA1 group, but no change was observed in mutated sequence, suggesting that UCA1 bound to miR-331-3p. Furthermore, miR-331-3p inhibited the luciferase activity in EIF4G 3′UTR-WT group, but not EIF4G1 3′UTR-MUT group in 22RV1 and DU145 cells (Fig. 6d, e). Besides, the expression of miR-331-3p was decreased in PCa tissues (Fig. 6f). Analogously, the low expression of miR-331-3p was found in 22RV1 and DU145 cells (Fig. 6g). Notably, the expression of miR-331-3p was inversely correlated with UCA1 level (Fig. 6h) or EIF4G1 level (Fig. 6i) in PCa tissues. Moreover, anti-miR-331-3p successfully restored the mRNA and protein expression of EIF4G1 in 22RV and DU145 cells transfected with si-UCA1 (Fig. 6j, k). The results of this part suggested that UCA1 could regulate the expression of EIF4G1 by targeting miR-331-3p.

## Discussion

LncRNA UCA1 was found to be aberrantly expressed in various cancer tissues [6, 7, 47] and multiple studies indicated that UCA1 worked as an oncogene in cancers. For instance, UCA1 deletion suppressed cell invasion and colony survival fraction, and induced cell cycle arrest by inhibiting EMT progression in colorectal cancer [48]. He et al. [49] demonstrated that UCA1 was upregultaed in PCa. Consistently, our data showed that the expression of UCA1 was increased in PCa tissues and cells. A previous research indicated that UCA1 knockdown contributed to increase the radiosensitivity of PCa cells in vitro [11]. In this study, we found that Gy irradiation increased the level of UCA1 in PCa cells. In addition, UCA1 knockdown enhanced the radiosensitivity of PCa cells by suppressing cell proliferation and promoting cell apoptosis.

Our data showed that EIF4G1 was increased in PCa tissues and cells. Moroever, EIF4G1 expression was positively related to UCA1 level in PCa tissues. Thus, we hypothesized that UCA1 regulated the radiosensitivity of PCa cells through regulating EIF4G1 expression. Previous studies reported that EIF4G1 was confirmed to be highly expressed and regulated the progression of various cancers, including PCa [35, 50]. However, the effect of EIF4G1 on the radioresistance of PCa cells is rarely explored. Our results suggested that EIF4G1 was upregulated by 6 Gy irradiation in PCa cells. Moreover, EIF4G1 was positively regulated by UCA1 in Gy irradiated PCa cells. Furthermore, we found that EIF4G1 deletion also elevated the radiosensitivity of PCa cells. Therefore, we investigated whether UCA1 mediated the radiosensitivity of PCa cells by regulating EIF4G1 expression. As expected, our findings suggested that UCA1 knockdown accelerated the radiosensitivity of PCa by inhibiting EIF4G1 expression.

MiRNAs were reported to be involved in the progression of PCa [51–53]. LncRNAs functioned as molecular sponges to competitively bind to the target miRNA, thereby achieving the regulation of target genes. For example, circ-0001649/miR-331-3p axis could be regarded as a potential target for non-small cell lung cancer therapy [54]. Zhang et al. [32] suggested that circ-CACTIN acted as a sponge for miR-331-3p to contribute to cell proliferation, migration, invasion, and EMT in gastric cancer by regulating TGFBR1 expression. Thus, we explored whether EIF4G1 directly regulated the biological function of UCA1. Our data indicated that miR-331-3p was a target of UCA1 and directly regulated the expression of EIF4G1. Taken together, our findings demonstrated that UCA1 regulated the radiosensitivity of PCa cells by sponging miR-331-3p to regulate EIF4G1 expression.

## Conclusions

We concluded that suppression of UCA1 facilitated the radiosensitivity in PCa by inhibiting EIF4G1 expression via miR-331-3p, providing a potential therapeutic target for PCa.

## Data Availability

The analyzed data sets generated during the present study are available from the corresponding author on reasonable request.
